# Does bike-sharing reduce traffic congestion? Evidence from three mega-cities in China

**DOI:** 10.1371/journal.pone.0306317

**Published:** 2024-08-20

**Authors:** Xiaoxia Xu, Wenbin Zuo

**Affiliations:** School of Economics, Jinan University, Guangzhou, China; The University of Tokyo, JAPAN

## Abstract

This study employs a regression discontinuity design to systematically examine the governance effect of bike-sharing on urban traffic congestion, utilizing city-level data from Beijing, Shanghai, and Wuhan in China between 2016 and 2018. We discover that the introduction of bike-sharing services significantly mitigates traffic congestion in the short term. Our heterogeneity analysis reveals that the initial deployment of shared bicycles primarily alleviates urban congestion, while additional deployments have a limited impact. Further, mechanism test analysis demonstrates that bike-sharing leads to increased metro ridership in these cities, effectively explaining the reduction in road congestion. This study underscores the pivotal role of bike-sharing services in easing urban traffic congestion and provides vital policy insights for enhancing traffic management strategies in Chinese cities.

## 1. Introduction

Road traffic congestion is a severe global problem [1]. After the rapid increase in motor vehicle ownership in the 1990s, numerous Chinese cities experienced severe traffic congestion problems, resulting in substantial social costs. Moreover, the accompanying exhaust emissions directly threaten the health of urban residents. According to the *China Mobile Source Environmental Management Annual Report 2019*, the number of cars in China reached 240 million in 2018. Urban traffic congestion generates large amounts of exhaust emissions, which are the largest contributors to pollutant emissions in China. Densely populated large cities, such as Beijing and Shanghai, have many buildings and congested roads [2], thereby impeding the dispersion of pollutants [3]. More than 20% of the PM2.5 concentrations in these cities come from automobiles, and their spread negatively affects the health of residents.

Given the above considerations, urban traffic congestion is related to an increase in the number of motor vehicles. Accordingly, how to manage traffic effectively and relieve road congestion has attracted global attention. Previous attempts to expand roads and impose congestion charges have failed to effectively solve congestion problems. In recent years, numerous countries have focused on promoting the use of positive means of transportation [4–6], and metropolitan cities have aimed to create an urban environment that is conducive to travel by public transport [7]. Managers and businesses collaborate to implement measures such as rail, public, and shared transportation to alleviate traffic congestion. These transportation modes are primarily governed by market dynamics, prioritizing voluntary participation over mandatory implementation. This strategy is expected to significantly reduce vehicle utilization in urban sectors, thereby effectively alleviating traffic congestion.

**Fig 1:**
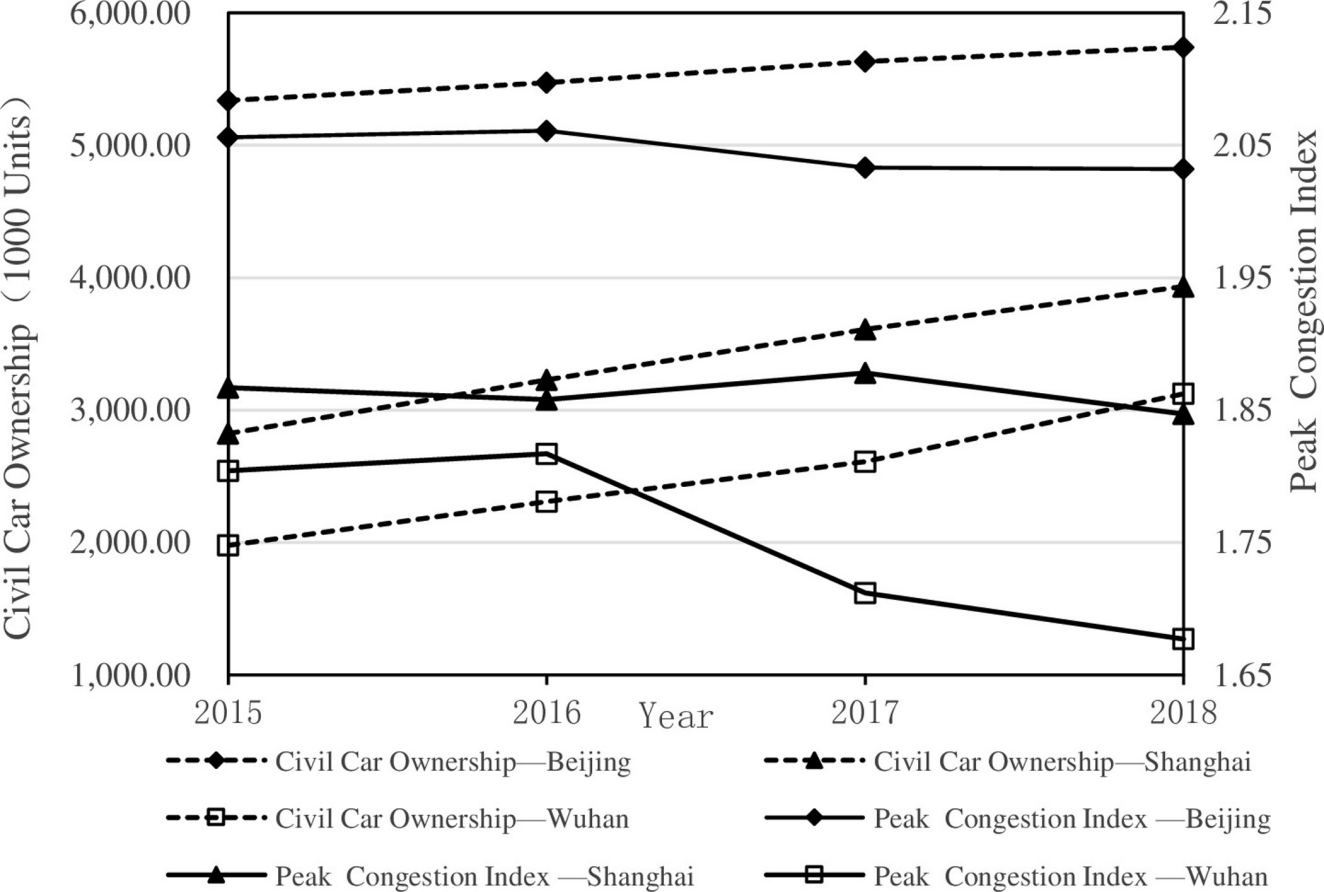
Civil car ownership and PCDI in Beijing, Shanghai, and Wuhan from 2015 to 2018.

**Fig 1** shows the number of civilian vehicles and the peak congestion delay index (PCDI) in Beijing, Shanghai, and Wuhan from 2015 to 2018. By denoting the ratio of peak travel time to travel time under free flow, PCDI has been used to measure traffic congestion. Another related measure is congestion delay index (CDI), which represents the ratio of the actual travel time of an average urban resident for one trip to the travel time in a free state. A higher value of these indices corresponds to a higher proportion of travel delay in travel time and a heavier traffic congestion. In Fig 1, it is evident that the number of private vehicles in these three cities has been increasing over the past four years. However, since 2016, the PCDI of these cities has shown a general downward trend. Notably, in 2016, bike-sharing services have emerged in these cities and quickly became one of the transportation infrastructures that connect various modes of travel. Since then, a new generation of bike-sharing services have gained popularity in Chinese cities, and bike-sharing has become the third-largest public travel mode in the country after buses and subways. Whether bike-sharing accounts for the reduction of motor vehicle ownership and consequently alleviates traffic congestion remains an unresolved question.

The effectiveness of bike-sharing in improving traffic has always been under scrutiny, especially due to its "stop-and-go" non-mandatory parking regulations. Despite the rise of bike-sharing in China in 2015, related research remains relatively scarce. Due to the limited traffic data in China and the difficulty in establishing causal relationships, the potential role of bike-sharing in urban traffic management lacks substantial empirical support. This limitation has hindered in-depth research into the effects of bike-sharing on improving transportation systems.

The primary objective of this research is to examine how bike-sharing impacts short-term transportation, specifically in terms of vehicle congestion in Beijing, Shanghai, and Wuhan. Given that the launch of bike-sharing resembles a natural experiment, we opt to employ a regression discontinuity design (RDD) for our study, as this method effectively mitigates estimation bias caused by endogeneity [8]. First, in our baseline regression, we focused on estimating the short-term effects of bike-sharing services 25 days before and after their introduction using a breakpoint regression. Our research findings indicate that bike-sharing significantly alleviates traffic congestion in the short term. The introduction of these services led to a substantial decrease of 12.253% in the average value of CDI. Second, we conduct robustness tests, which included bandwidth sensitivity testing, excluding interference from holidays, restricted driving policies, and extreme weather, as well as utilizing alternative regression models and conducting continuity checks for control variables. Across various subsamples, the results remained robust. In the subsequent heterogeneity analysis, we assess the impact of bike-sharing services over various years and among different bicycle manufacturers. We discover that the early deployment of bicycles by leading manufacturers substantially alleviates traffic congestion. Finally, our mechanistic analysis reveals that the launch of bike-sharing services leads to a significant increase in daily subway ridership, which emerges as a potential mechanism for alleviating road congestion. Given that the breakpoint regression empirical strategy relies on discontinuous changes in traffic congestion before and after the launch of bike-sharing services and on the continuous changes in other factors that influence traffic, our findings are only applicable to short-term scenarios.

This study makes several contributions: First, it supplements evidence from China by using a multi-cutoff RDD to explore the relationship between bike-sharing services and traffic congestion across different regions in China. While there has been extensive research on the impacts of railways and buses on traffic congestion [8–10], studies on bike-sharing in China remain relatively scarce. Direct investigations into the relationship between bike-sharing and traffic congestion are even more limited. Second, this study compares multiple bike-sharing brands in China, broadening the scope of previous research. Third, it provides a scientific basis and reference for the development and standardization of bike-sharing services, assessing their impact on alleviating traffic congestion. This evaluation is crucial as these services offer a low-cost alternative to subways and buses and help reduce the externality of automobiles in urban areas [11].

The rest of this paper is organized as follows. **Section 2** reviews the related literature, and **Section 3** introduces the data and econometric model. **Section 4** presents the empirical analysis, and **Section 5** concludes the paper and presents some policy implications.

## 2. Literature review

Previous research has categorized methods of managing traffic congestion into two types. The first includes traditional governance measures, driven by government-led initiatives and enforced through state authority. These measures typically involve expanding roads and imposing congestion charges. Studies indicate that increasing road capacity often leads to more driving, failing to effectively solve congestion [12, 13]. Additionally, research has demonstrated that congestion charges can effectively mitigate congestion [14]. But in practice, few cities have successfully implemented congestion charging, with its implementation facing challenges such as high costs and public resistance [15–17]. Therefore, these strategies alone are insufficient to significantly improve traffic congestion.

In recent years, a growing number of countries have redirected their attention towards an alternative solution, optimizing public transportation services to improve traffic congestion. These strategies, which rely on voluntary use by residents, include subway systems, buses, and bike-sharing programs. Research indicates that enhancing public transportation efficiency can reduce private vehicle use and alleviate congestion. For instance, Mulalic and Rouwendal (2020) found that better public transport in the Copenhagen area significantly lowered private car ownership rates [18]. In Xi’an, China, Huang et al. (2019) observed that the subway reduced individual car travel, effectively easing urban congestion [19]. Similarly, Yang et al. (2018) reported that the opening of six subway lines in Beijing between 2009 and 2015 significantly reduced short-term traffic congestion [10]. Disruptions in public transport, like bus strikes studied by Nguyen-Phuoc et al. (2018), can increase private vehicle use, worsening congestion [5]. Anderson (2014) found that road congestion increases by 47% when bus services are interrupted [8]. Beaudoin et al. (2015) concluded that investments in public transportation not only improve traffic flow but also enhance environmental quality [11]. These findings underscore the efficacy of public transportation improvements in addressing urban congestion.

Following subways and buses, bike-sharing has recently become one of the most used modes of transportation in China. Since the emergence of the first-generation bike-sharing program in Amsterdam in 1965 [20], shared “white bicycles” have undergone five generations of system updates. In 2016, dockless bike-sharing platforms launched in China, initiating the first market-driven fifth generation of bike-sharing and sparking a global expansion [21]. The fifth generation of bike-sharing supports GPS tracking and QR-code unlocking, enabling dockless use. It is operated by companies, regulated by the government, and market driven. Although bike-sharing has been around for a long time, significant academic interest in this area only began to develop after 2010. Research has primarily concentrated on several key areas: the factors and barriers influencing bicycle usage [22–28], the expansion and optimization of these systems [29–31], and the cycling behaviors of users along with their impacts [32–34]. As a low-carbon and environment-friendly transportation method, bike-sharing provides many benefits, including but not limited to alleviating road traffic congestion and improving air quality [20, 32, 35].

The primary goal of bike-sharing services is to reduce reliance on automobiles, thereby easing traffic congestion and enhancing air quality [35]. The debate on whether bike-sharing significantly mitigates traffic congestion remains unresolved. Economic theories contend that these services have uncertain impacts. As a synergistic mode of travel, an individual who rides a shared bike to the bus or subway is equivalent to one less car user, thereby contributing to road decongestion. In Washington, D.C., Ma et al. (2015) and Hamilton and Wichman (2018) found that bike-sharing reduces congestion, particularly in crowded areas, by complementing public transportation [36, 37]. In China, since 2015, bike-sharing has rapidly developed into a new environmentally mode of transportation, attracting considerable academic interest [38]. Qin et al. (2018) used an RDD approach and discovered that introducing bike-sharing platforms significantly decreased short-distance ride-sharing orders in Chengdu, China [39]. Ma et al. (2019) also found evidence in Chengdu that bike-sharing contributes to an increase in public transit ridership [40]. Using a difference-in-differences approach, Fan and Zheng (2020) analyzed daily passenger volumes on 10 Beijing subway lines before and after bike-sharing was introduced [41]. They found that subway lines with more bike-sharing had increased ridership and less congestion around stations during peak times. Similarly, X.-H. Yang et al. (2018) found that bike-sharing in Hangzhou and Ningbo reduced the number of transfers and commute times [42]. These studies consistently demonstrate that bike-sharing effectively increases subway and bus ridership, thereby alleviating traffic congestion.

However, according to the law of peak-hour traffic congestion [43], traffic congestion continues to worsen until reaching the capacity of the roads. This happens because drivers are quickly drawn to less congested roads, replacing the impact caused by bike-sharing. Moreover, bike-sharing may occupy lanes on roads, thereby exacerbating traffic congestion. Studies by Wang and Zhou (2017) and Campbell and Brakewood (2017) suggest that the impact of bike-sharing on overall traffic reduction is limited and can even reduce bus ridership, potentially shifting rather than eliminating congestion [44, 45]. Moreover, the rapid expansion of bike-sharing systems has introduced new challenges, such as parking congestion [46]. The debate on the role of bike-sharing in alleviating urban traffic congestion continues, highlighting the complex impact of bike-sharing on urban transportation systems.

In China, the bike-sharing market emerged in 2015. By the end of 2017, more than 20 companies had either shut down or been acquired. Additionally, each bike-sharing enterprise holds its operational data, resulting in limited data available for research. The potential role of bike-sharing in urban traffic management lacks substantial empirical support. Existing studies have only indirectly explored the possible impacts of bike-sharing on traffic congestion through changes in bus or subway ridership. However, this indirect approach is insufficient to fully understand the effects on traffic congestion. According to the law of peak-hour traffic congestion theory [43], newly expanded roads immediately attract new vehicles until saturation reoccurs. To address these gaps, this study employs a multi-cutoff RDD to directly investigate the impacts of bike-sharing services on the urban transportation system.

## 3. Data source and model setting

### 3.1 Data source

Our research samples were collected from 2016 to 2018 in three major Chinese cities—Beijing, Shanghai, and Wuhan—specifically chosen for their high levels of congestion. According to AutoNavi’s *2015 Traffic Analysis Report of Major Cities in China*, Beijing has a Peak Congestion Delay Index (PCDI) of 2.06, indicating that commuters spend twice as long traveling during peak hours compared to less congested times, which reflects the highest congestion costs in the nation. Our study focuses on the impact of bike-sharing on traffic congestion in these cities, and the insights may be applicable to other highly congested cities. Additionally, the *China Statistical Yearbook* reports that by the end of 2018, Beijing and Shanghai, with populations exceeding 20 million, were among China’s ten most populous cities. Mobile emissions are a primary source of PM2.5 pollution in these cities. Exploring the intersection of bike-sharing and traffic congestion could provide valuable strategies for reducing environmental pollution in these and other densely populated urban areas.

Our study focused on six leading bike-sharing companies: Mobike, OFO, Youon, Bluegogo, Green Orange Bike, and Hellobike. Originally designed to enhance transportation options for university students, bike-sharing evolved rapidly when Mobike and OFO launched dockless bicycles in 2015, signaling the advent of market-oriented bike-sharing in China. This innovation quickly extended beyond educational campuses, with Mobike securing considerable investment by October of the same year. By 2017, according to the "*China Bike-Sharing Industry Development Report (2018)*" by *China Academy of Information and Communications Technology* (CAICT), Mobike and OFO together controlled 78.3% of the market share. Meanwhile, other companies, including Bluegogo and Youon, entered the market and quickly expanded across China’s tier 1 and tier 2 cities, becoming significant players. Despite challenges like financial difficulties leading to restructuring in companies like OFO, the industry’s influence remained considerable. OFO holds such a large market share that new entrants are likely to simply refurbish and redeploy these renovated old bicycles. Bike company logos change however, the number of bikes stays the same.

From April 22, 2016 to March 22, 2018, 6 major market-based bike-sharing services have appeared in Beijing, Shanghai, and Wuhan, namely, Mobike, OFO, Youon, Bluegogo, Green Orange Bike, and Hellobike. The specific dates of their launch are shown in **Table 1**. Bike sharing service providers usually make media announcements on their official websites when they implement bike sharing in a city, and some official news is also reported. We hand-collected and organized data on the launch date of the bike-sharing service. For instance, information was sourced from OFO’s official Sina Weibo page: https://weibo.com/5122220173/EcegNzyHj.

**Table 1:** Launch dates of bike-sharing services in Beijing, Shanghai, and Wuhan from 2016 to 2018.

City Shared Bicycles	Beijing	Shanghai	Wuhan
Mobike	2016.8.16	2016.4.22	2016.12.29
OFO	2016.10.11	2016.10.10	2017.1.6
Youon	2017.2.21	2017.2.21	
Bluegogo	2017.2.21		
Green Orange bike			2018.3.22
Hellobike			2017.3.11

Notes: The blanks in the table indicate that the corresponding bike-sharing providers are not available in the city or are yet to be acquired.

Data on the primary explained variable, CDI, were collected from the WIND database (https://www.wind.com.cn/). The WIND database is one of China’s leading providers of analytical tools and financial data, and it contains daily CDI indices for cities from 2016–2018. CDI is used as an evaluation index for the degree of urban congestion, that is, the ratio of urban residents’ average travel time to their travel time under the free flow state. A higher CDI corresponds to a more significant travel delay proportion in the total travel time and a higher traffic congestion.

Our main control variables include extreme weather (i.e., temperature, precipitation, and wind speed), traffic restrictions, DNvalue, population density, and length of subway and bus lines. Our data on extreme weather were collected from the *China Meteorological Science Data Sharing Service Network* (www.data.cma.cn). Extreme weather includes heat waves, cold snaps, heavy rains, strong winds, and heavy snowfall [10]. A heat wave is observed when the maximum temperature exceeds 35°C for more than three consecutive days. A cold snap occurs when the lowest temperature drops below 4° C. Heavy rains are reported when the amount of rainfall reaches 50 mm or more in 24 hours. Gales are eight-level-intensity winds with speeds ranging from 17.2 o 20.7 m/s. A heavy snowfall is observed when the amount of snow exceeds 5.0 mm in 24 hours. We collect our data on traffic restrictions from the announcements of the *Urban Transportation Bureau* (https://www.mot.gov.cn/) and our data on night-time light DNvalue from the *National Oceanic and Atmospheric Administration* (NOAA) (http://ngdc.noaa.gov/eog/dmsp/downloadV4composites.html). We used the night-time light DNvalue to indicate the economic development level of the three selected cities. We obtained our data on population density and operating length of subways and buses from the *National Statistical Yearbook* (http://www.stats.gov.cn/sj/zgtjnj/) and the *Urban Statistical Yearbooks* of the three selected cities from 2016 to 2019 (https://nj.tjj.beijing.gov.cn/; https://tjj.sh.gov.cn/; http://tjj.wuhan.gov.cn/). For variables with missing values, we use the average of the values from the previous and subsequent periods to fill these gaps.

### 3.2 Model setting

To assess the relationship between bike-sharing and traffic congestion, we employed a RDD strategy for several reasons. First, the challenge of endogeneity and the difficulty of creating counterfactual samples render the ordinary least squares (OLS) method susceptible to methodological flaws, often leading to inconsistent estimates in simple OLS regression. Second, unlike OLS, RDD is a quasi-experimental approach that approximates randomized trials in terms of causal inference and internal validity, allowing causal conclusions to be drawn even in the absence of random assignment [47]. RDD provides a natural exogenous pathway for studying local effects. The unique advantage of RDD in addressing endogeneity issues has led to its continuous refinement and use by some econometricians [48–50].

The use of RDD in economic and social research requires certain conditions: first, there must be a sudden policy event creating a natural breakpoint; second, other control variables near the breakpoint must remain continuous and not undergo sudden changes. Given the clear time cutoff point for the introduction of new shared bicycles, this can be seen as a factor of mutation; therefore, sharp RDD were used in this study. If a noticeable shift in the Congestion Delay Index (CDI) occurs following the bike-sharing rollout, it indicates a direct correlation, considering the constancy of other variables. We build a temporal multi-breakpoint regression model given that multiple providers correspond to various entry times. The specific RDD model is as follows:

$$CDI_{it}=\beta_{0}+\beta_{1}\;Bike\_sharing_{it}+\beta_{2}\;f(T)+\beta_{3}\;Bike\_sharing_{it}f(T)+\lambda X_{it}+\mu_{it}$$ (1)

$$f(T)=a_{0}+a_{1}T+a_{2}T^{2}+\cdots +a_{n}T^{n}$$ (2)

where the explained variable *CDI*_*it*_ represents the CDI of city *i* on date *t*, *Bike_sharing*_*it*_ is a dummy variable indicating whether bike-sharing services have been launched on date *t* in city *i*, and its regression coefficient *β*_1_ is the value of interest in this article, which captures only the changes in the congestion index before and after the launch of bike-sharing services. *T* is a running variable that represents the number of days from the launch of bike-sharing services. This value takes a value of 0 on the launch day, a negative value before the launch day, and a positive value after the launch day. The number of days before and after the launch of bike-sharing services presents the bandwidth for determining the range of the local regression. *f* (*T*) represents the polynomial of *T*. It allows for a more flexible relationship between T and *CDI*, capturing non-linear effects. The regression model also controls for those variables that affect the traffic patterns of *X*_*it*_, including the dummy variables of extreme weather, vehicle restrictions for the three cities on date *t*, night-light DNvalue, population density, and operating length of subways and buses.

The local polynomial method involves selecting a kernel function, bandwidth, and the polynomial degree. We used a triangular kernel function, which gives maximum weight near the breakpoint and decreases linearly with distance. Due to the close entry times of various bike-sharing companies, some dates in the sample were classified into the treatment group at one breakpoint and into the control group at the next. To address this issue of sample grouping, we implemented two solutions: First, we set the bandwidth for the baseline regression at 25 days and expanded it in subsequent tests. Second, we divided the sample into two parts, calculated the average between the two breakpoints, then assigned the samples near the previous breakpoint to the treatment group and those near the next breakpoint to the control group, assigning them negative values [51]. Based on the bandwidth and trend graphs, we focused on quadratic and cubic polynomial regressions.

## 4. Empirical results and analysis

This section empirically tests the impact of bike-sharing on CDI. The first subsection describes the statistical analysis, the second subsection uses RDD for the benchmark regression, the third subsection presents the robustness checks and endogeneity analysis, the fourth subsection discusses the heterogeneity test and the fifth examine the mechanism analysis.

### 4.1 Statistical analysis

The descriptive statistical characteristics of the explained variables, core explanatory variables, and control variables are shown in **Table 2**. The observations cover the data of 25 days before and after the launch of bike-sharing services in Beijing, Shanghai, and Wuhan from June 22, 2016, to May 16, 2018.

**Table 2:** Description of variables.

Variable	Observations	Mean	Std. Dev.	Min	Max
CDI	967	1.738	0.313	1.07	3.27
Extreme Weather	967	0.291	0.454	0	1
Traffic Restriction	967	0.45	0.498	0	1
DNvalue	967	9.567	4.234	4.71	17.74
Population Density	967	2238.846	1217.768	1256.39	3816.26
Length of line in operation	967	18659.18	6206.993	9138.7	24798.3

Data source: CDI data are compiled from the WIND database (https://www.wind.com.cn/). Extreme weather data are obtained from the *China Meteorological Science Data Sharing Service Network* (www.data.cma.cn). Traffic restrictions data are taken from the traffic policy announcements issued by the *Transportation Bureaus* of the three cities (https://www.mot.gov.cn/). Night-time light DNvalue data are collected from the *National Oceanic and Atmospheric Administration* (NOAA) (http://ngdc.noaa.gov/eog/dmsp/downloadV4composites.html). Data on population density and operating length of subways and buses are derived from the *National Statistical Yearbook* and the *Urban Statistical Yearbook* of the three cities for the years 2016 to 2019 (http://www.stats.gov.cn/sj/zgtjnj/).

**Fig 2:**
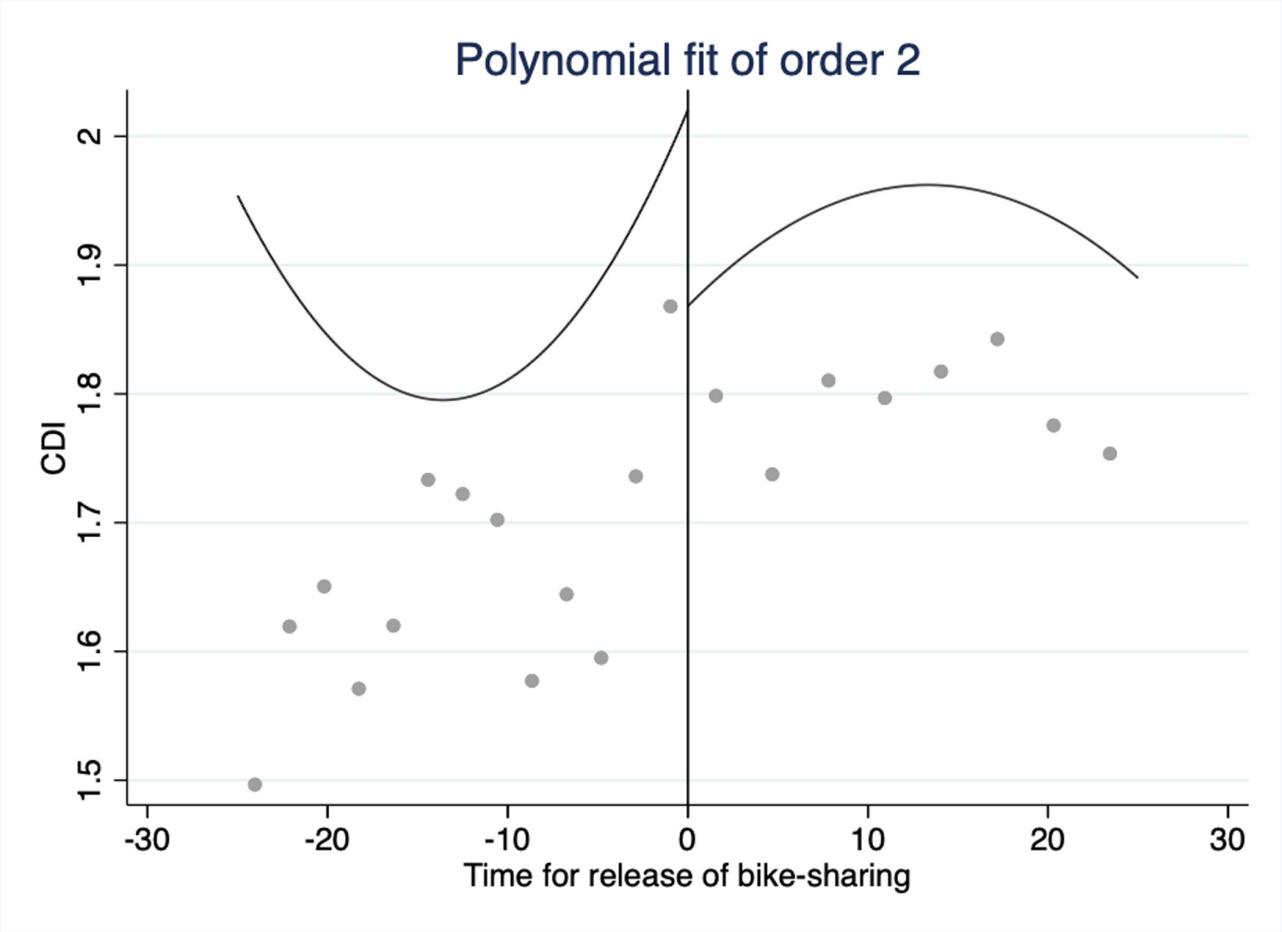
Second-order polynomial fit, where the 25 days before and after the entry of bike-sharing services are set as the bandwidth.

**Fig 3:**
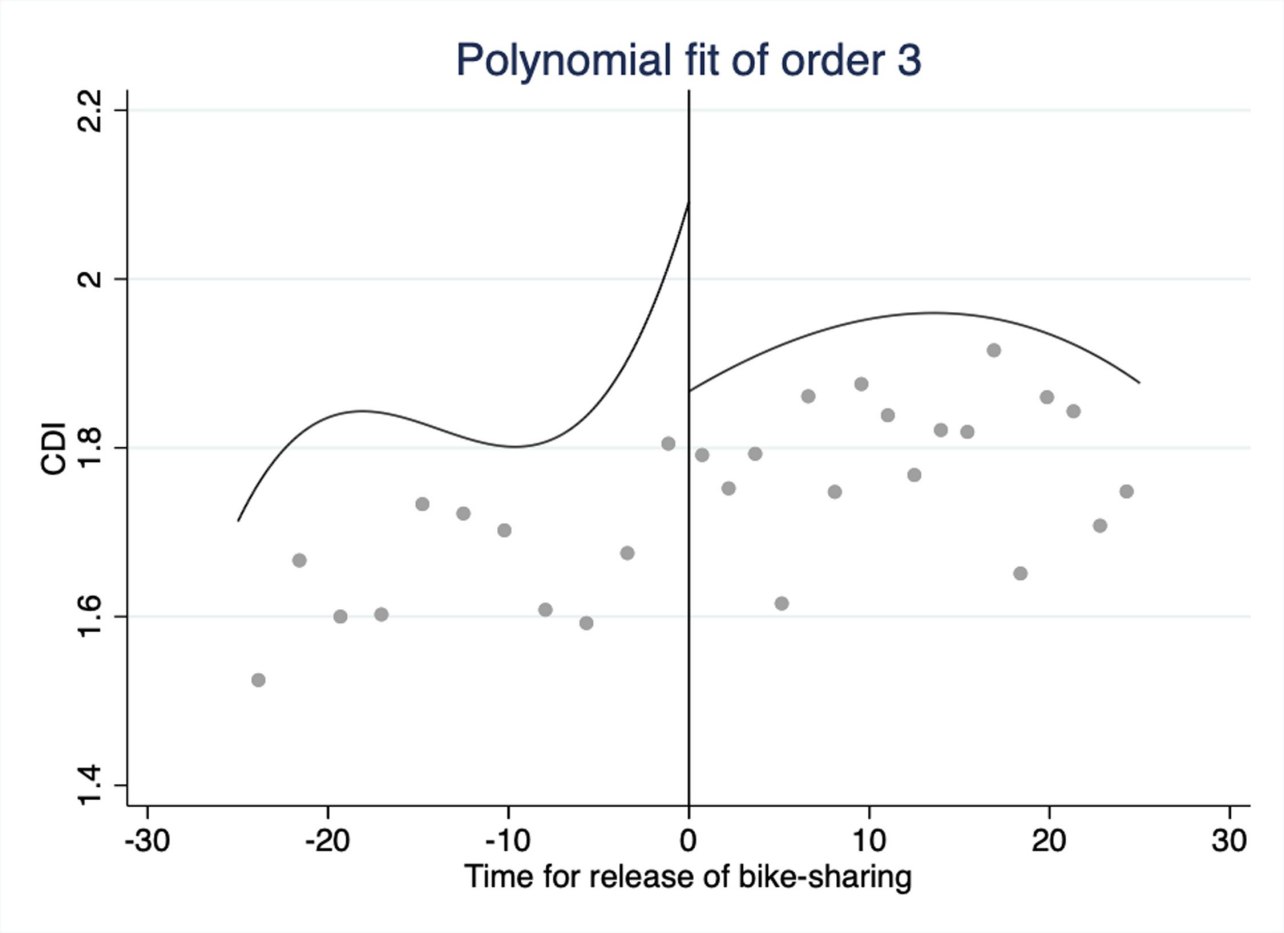
Third-order polynomial fit, where the 25 days before and after the entry of bike-sharing services are set as the bandwidth.

Taking the launch date of bike-sharing services as the breakpoint, we draw the fitting curve of the CDI for 25 days before and after the launch of these services. **Figs 2** and **3** employ second-order and third-order polynomials, respectively, to draw the CDI fitting curve. The CDI demonstrates an overall upward trend before the breakpoint but a downward trend after the breakpoint. The trend from the graph below reveals the initial relationship between bike-sharing and traffic congestion. We focus on the results of the third-order polynomial. Compared with higher-order polynomials, third-order polynomials give more weight to the samples near the breakpoint [10, 52]. This functional form allows for nonlinearities in transport behavior as long as these trends are continuous. First-order polynomials are not appropriate because the dependent variable exhibits nonlinearities. However, for rigorous considerations, we also include the results of the second-order polynomials in our later analysis.

### 4.2 Benchmark regression results

Based on 25 days before and after the launch of bike-sharing services, we use the above formula to estimate the relationship between bike-sharing and traffic congestion. **Table 3** presents the results with the controls. The regression results indicate that the launch of bike-sharing services has a mitigating effect on CDI. Columns (1) to (3) include the covariates that may affect travel patterns, including the dummies of travel restrictions and extreme weather and the control variables of DNvalue, population density, and length of subway and bus lines. The parameter under the third-order polynomials in column (3) indicates that the CDI is significantly mitigated by 0.226 following the introduction of bike-sharing services. This estimate is equal to 13% of the average CDI value. In addition, robust standard errors are used in all regressions in the text. Robust standard errors are insensitive to possible heteroskedasticity and autocorrelation problems in the model, thereby improving estimation accuracy [53].

**Table 3:** Effect of bike-sharing on CDI.

Dependent variable:	CDI		
	(1)	(2)	(3)
	Linear	Binomial	Trinomial
**Bikesharing**	-0.023	-0.132^***^	-0.226^***^
	(0.038)	(0.056)	(0.068)
Restriction	Y	Y	Y
Extreme Weather	Y	Y	Y
DNvalue	Y	Y	Y
Population Density	Y	Y	Y
Length of line in operation	Y	Y	Y
Observations	463	463	463

Notes: The table covers the period 25 days before and after the launch of bike-sharing services. We include week fixed effects in all columns. In columns (1) to (3), we include those covariates that may affect travel patterns, namely, the dummies of travel restrictions, extreme weather, weekends, and control variables of DNvalue, population density, and length of subway and bus lines. Robust standard errors are reported in parentheses; *** p<0.01, ** p<0.05, and * p<0.1.

### 4.3 Robustness test and endogeneity analysis

Although the empirical results in **Table 3** preliminarily reveal the mitigating effect of bike-sharing on traffic congestion, the effectiveness of RDD may be limited by other conditions. We then conduct a robustness test in this section.

#### 4.3.1 Adjust the bandwidth

The bandwidth of the RDD influences the validity of the estimation results. Specifically, a smaller bandwidth decreases bias but increases variance due to the limited number of observations, while a larger bandwidth incorporates more observations, reducing variance but increasing bias. For the baseline regression, the bandwidth is set at 25 days. To test for robustness, we adjust this bandwidth to two months (60 days) and half a month (15 days) before and after the introduction of bike-sharing services.

Panels A and B in **Table 4 s**how the results for the third- and second-order polynomials, respectively. In Panel A, we control for those factors that may affect traffic patterns, including traffic restrictions, extreme weather, DNvalue, population density, and length of subway and bus lines. Overall, bike-sharing has a significant mitigating impact on CDI, thereby supporting the robustness of the empirical conclusions in **Table 3**. The magnitude and significance level of the coefficient estimate for shared bike entry decreases with increasing bandwidth, which seems to indicate that the impact of shared bikes on reducing urban congestion is more significant in the short run than in the long run. Some drawbacks slowly unfold over time. This may imply that subsequent factors, such as poor operational management, lead to a reduction in effectiveness.

**Table 4:** Bandwidth sensitivity test.

Dependent variable:			CDI			
**Panel A**	**Trinomial**					
Bandwidth:	(1)	(2)	(3)	(4)	(5)	(6)
	15	20	30	40	50	60
**Bike sharing**	-0.291 ^***^	-0.290^***^	-0.200^***^	-0.131^**^	-0.108^**^	-0.089^*^
	(0.079)	(0.072)	(0.066)	(0.060)	(0.054)	(0.05)
Restriction	Y	Y	Y	Y	Y	Y
Extreme Weather	Y	Y	Y	Y	Y	Y
DNvalue	Y	Y	Y	Y	Y	Y
Population Density	Y	Y	Y	Y	Y	Y
Length of line in operation	Y	Y	Y	Y	Y	Y
Observations	283	373	548	689	828	967
**Panel B**			**Binomial**			
	(7)	(8)	(9)	(10)	(11)	(12)
Bandwidth:	15	20	30	40	50	60
**Bike sharing**	-0.231^***^	-0.167^***^	-0.098^*^	-0.068	-0.042	-0.014
	(0.065)	(0.061)	(0.052)	(0.046)	(0.041)	(0.038)
Restriction	Y	Y	Y	Y	Y	Y
Extreme Weather	Y	Y	Y	Y	Y	Y
DNvalue	Y	Y	Y	Y	Y	Y
Population Density	Y	Y	Y	Y	Y	Y
Length of line in operation	Y	Y	Y	Y	Y	Y
Observations	283	373	548	689	828	967

Notes: We include week fixed effects in all columns. All columns include covariates that may affect travel patterns, namely, the dummies of travel restrictions, extreme weather, and the control variables of DNvalue, population density, and length of subway and bus lines. Panel A presents the results of the third-order polynomial with bandwidth (i.e., 15, 20, 30, 40, 50, and 60 days) before and after the bike-sharing launch. Panel B reports the results of the second-order polynomial. The bandwidth (i.e., 15, 20, 30, 40, 50, and 60 days) is reduced and extended from the 25-day bandwidth in the benchmark regression. Robust standard errors are reported in parentheses; *** p<0.01, ** p<0.05, and * p<0.1.

#### 4.3.2 Statutory holiday effect

The effect of bike-sharing on traffic congestion may bias the estimation results when holidays, such as the National Day, are captured. Therefore, in **Table 5**, we test the results of controlling for and eliminating the statutory holidays variable 25 days before and after the launch of bike-sharing services. The results are presented in columns (1) to (4) of **Table 5**. Columns (1) to (2) shows that when legal holidays are controlled for and when the bandwidth is set to 25 days, the parameter results of third-order polynomials indicate that the launch of bike-sharing services significantly reduces the CDI by 0.14 (8.06% of the average CDI value). In column (3)-(4), we exclude the holiday data and use the subsample for estimation. Results show that the entry of bike-sharing services significantly reduces the CDI by 9.95% of its average value as reported in column (4). These results support our argument that some reductions in CDI are due to holidays. Despite the considerable decrease in magnitude and significance compared with the baseline regression result (13% of the average CDI value), our findings remain robust.

**Table 5:** Holiday effect.

Dependent variable:	CDI			
Subsample	All sample		Excluding Holiday = 1	
	(1)	(2)	(3)	(4)
Bandwidth:	25		25	
	Binomial	Trinomial	Binomial	Trinomial
**bike sharing**	-0.104^**^	-0.140^**^	-0.145^**^	-0.173^**^
	(0.051)	(0.063)	(0.058)	(0.069)
Holiday	Y	Y	Excluding	Excluding
Restriction	Y	Y	Y	Y
Extreme Weather	Y	Y	Y	Y
DNvalue	Y	Y	Y	Y
Population Density	Y	Y	Y	Y
Length of line in operation	Y	Y	Y	Y
Observations	463	463	410	410

Notes: We include week fixed effects in all columns. All columns include extra covariates that may affect travel patterns, including DNvalue, population density, and length of subway and bus lines in operation. In columns (5) to (8), “N” indicates the exclusion of data on statutory holidays. Robust standard errors are reported in parentheses; *** p<0.01, ** p<0.05, and * p<0.1.

#### 4.3.3 Replacing metric models

In order to further validate the robustness of our findings, we employed a fixed effects model to estimate the relationship between bike-sharing and traffic congestion. Specifically, we used the initial deployment date of bike-sharing in each city as the basis for establishing dummy variables. Regardless of the brand of bike-sharing, as long as bike-sharing was initially introduced in a city, the dummy variable was set to 1 from that date onwards. For example, if Beijing introduced its first batch of bike-sharing on August 16, 2016, the dummy variable " bike-sharing " would be 0 before August 16, 2016, and 1 from August 16, 2016, onwards.

In the column (1) of **Table 6,** we employed "bike-sharing" as the explanatory variable and "CDI" as the explained variable, estimating the model over a sample period of 60 days while incorporating relevant control variables and weekly fixed effects. The results displayed in the first column reveal that the coefficient for the bike-sharing dummy variable stands at -0.035, achieving statistical significance at the 5% level. No notable deviations in either the sign or significance are observed when compared to the baseline regression or the results under the same bandwidth. This consistency lends robust support to our conclusions.

**Table 6:** Travel restrictions, extreme weather, and weekend effects.

Dependent variable:		CDI			
	(1)	(2)	(3)	(4)	(5)
Subsample:	OLS	Excluding Restriction = 1		Excluding Extreme Weather = 1	
		Binomial	Trinomial	Binomial	Trinomial
**bike sharing**	-0.035**	-0.158^*^	-0.252 **	-0.12	-0.233^***^
	(0.015)	(0.085)	(0.111)	(0.074)	(0.92)
Restriction	Y	Excluding	Excluding	Y	Y
Extreme weather	Y	Y	Y	Excluding	Excluding
DNvalue	Y	Y	Y	Y	Y
Population Density	Y	Y	Y	Y	Y
Length of line in operation	Y	Y	Y	Y	Y
Observations	967	270	270	338	338

Notes: We include week fixed effects in all columns. All columns include extra covariates that may affect travel patterns, including the control variables of DNvalue, population density, and length of subway and bus lines in operation. “N” indicates the exclusion of data on motor vehicle restrictions and extreme weather. Robust standard errors are reported in parentheses; *** p<0.01, ** p<0.05, and * p<0.1.

#### 4.3.4 Restrictions and extreme weather effects

Traffic restrictions are a significant factor influencing the choice to use motor vehicles, while extreme weather is an important factor affecting whether to ride bicycles. In this section, we set the bandwidth to 25 days before and after the launch of bike-sharing in Table 6. We then evaluate the robustness of our results by sequentially eliminating data on implemented restrictions and extreme weather. Columns (2)—(5) present the results. Compared with the baseline results in Table 3, the coefficients do not change much statistically and in magnitude, thereby supporting the robustness of our findings.

#### 4.3.5 Placebo test

RDD requires testing the continuity of the controls. The estimation effect of bike-sharing may be biased by capturing the influence of the control variables when they jump at the breakpoints. Consequently, if the variables vary gradually around the bike-sharing launch date, then the regression interruption approach removes the source of endogeneity outlined in the OLS approach.

**Fig 4:**
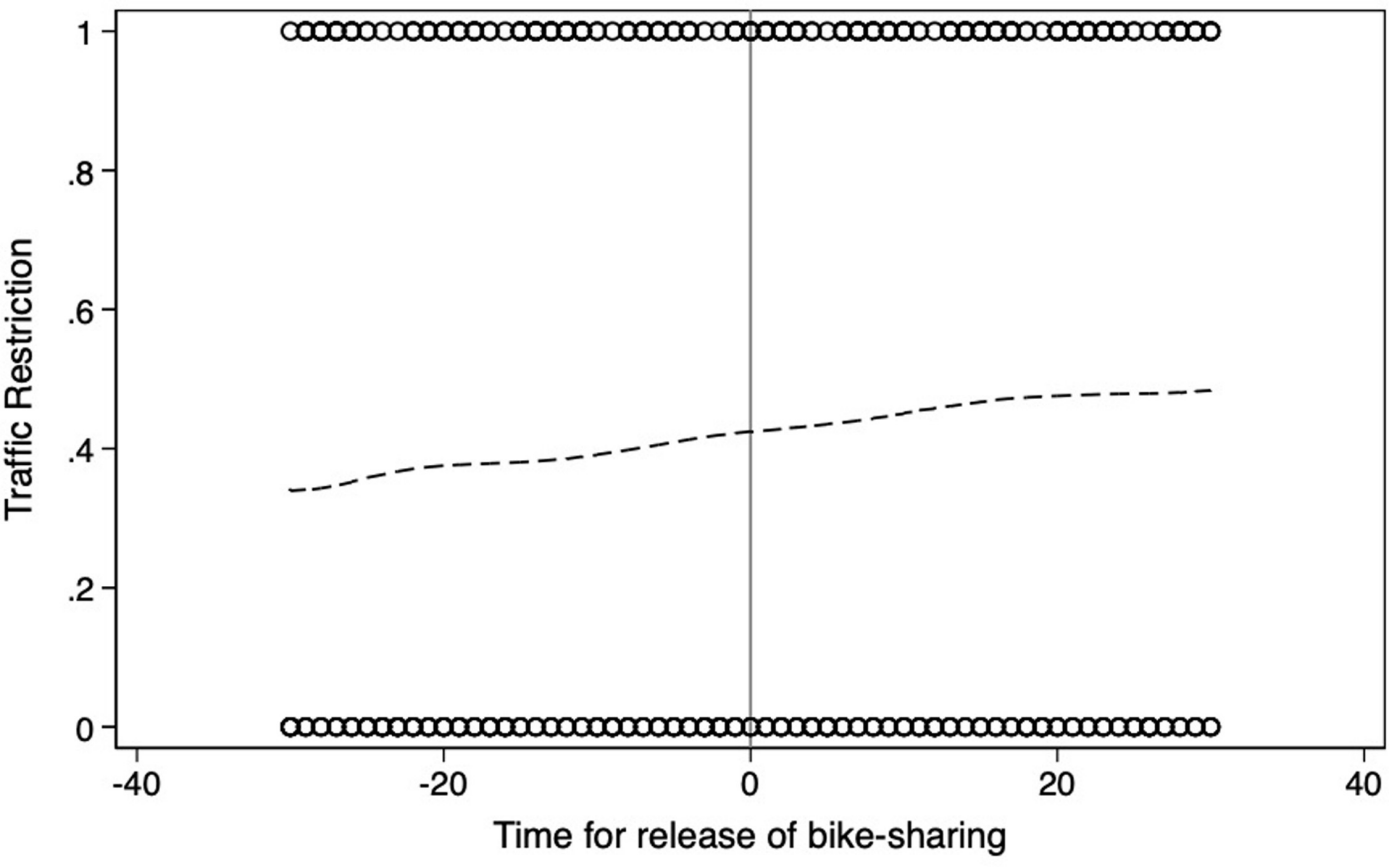
Trends of traffic restrictions for 25 days before and after the launch of new bike-sharing services.

**Fig 5:**
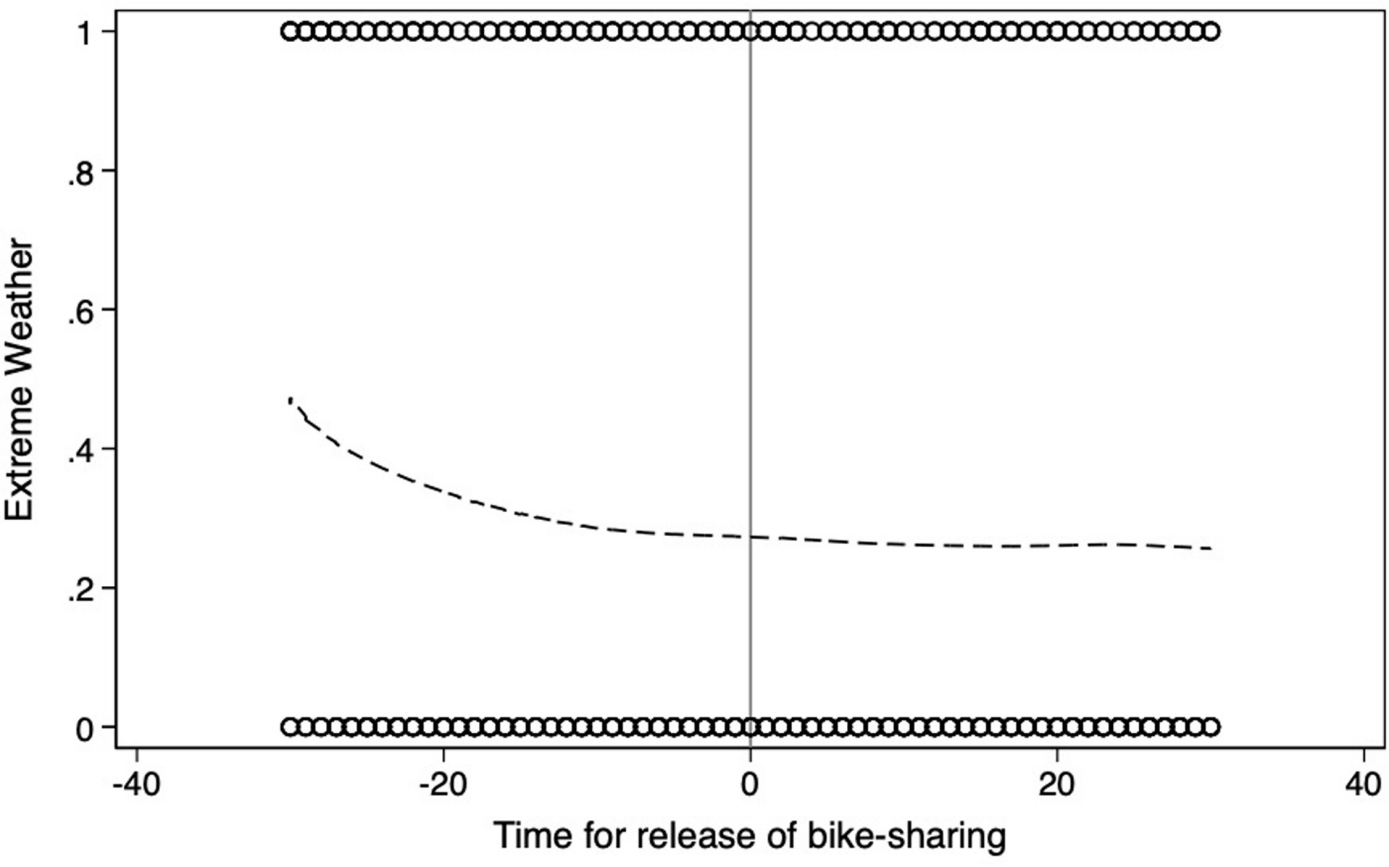
Trends of extreme weather for 25 days before and after the launch of new bike-sharing services.

**Fig 6:**
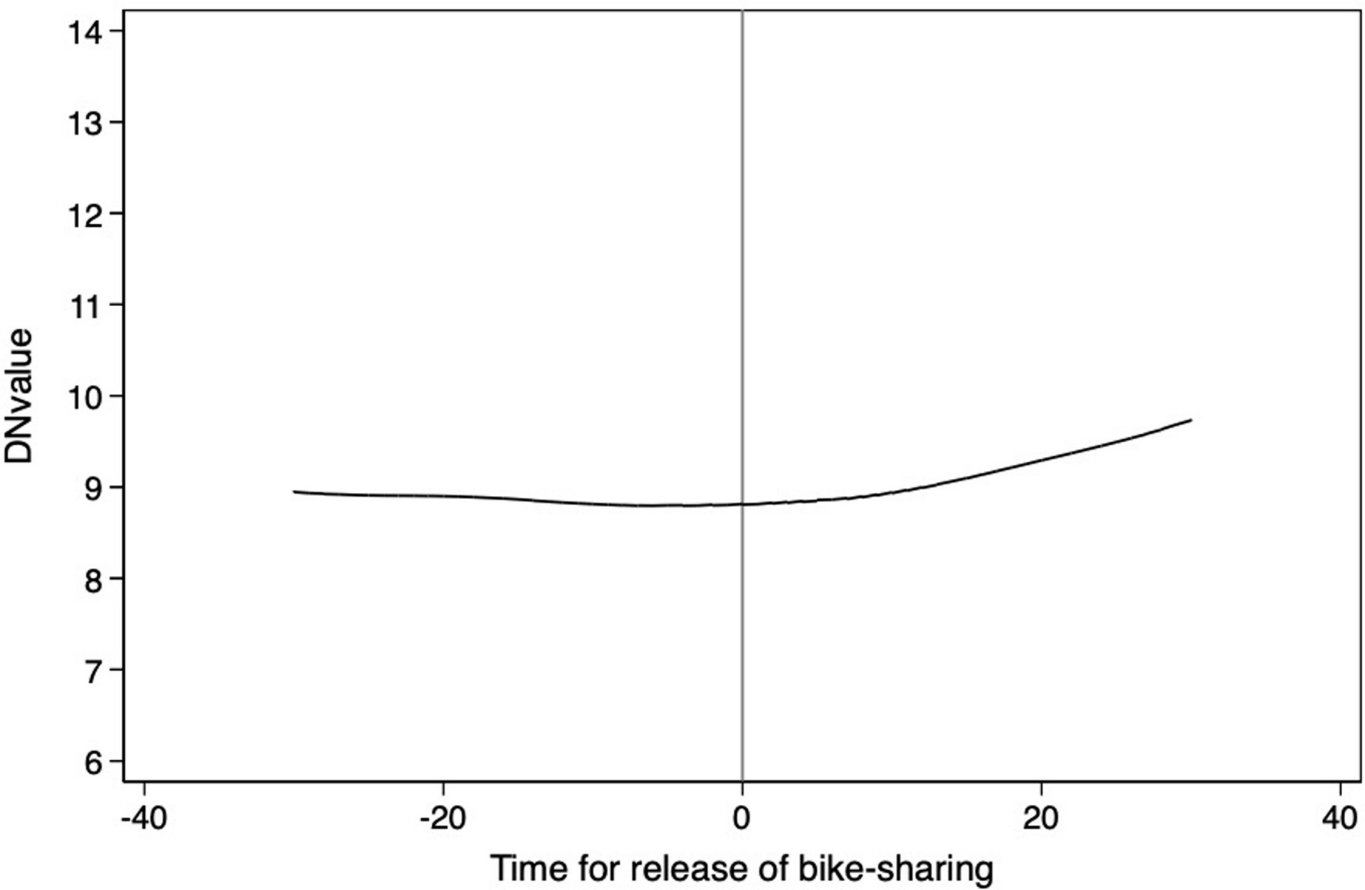
Trends of DNvalue for 25 days before and after the launch of new bike-sharing services.

**Fig 7:**
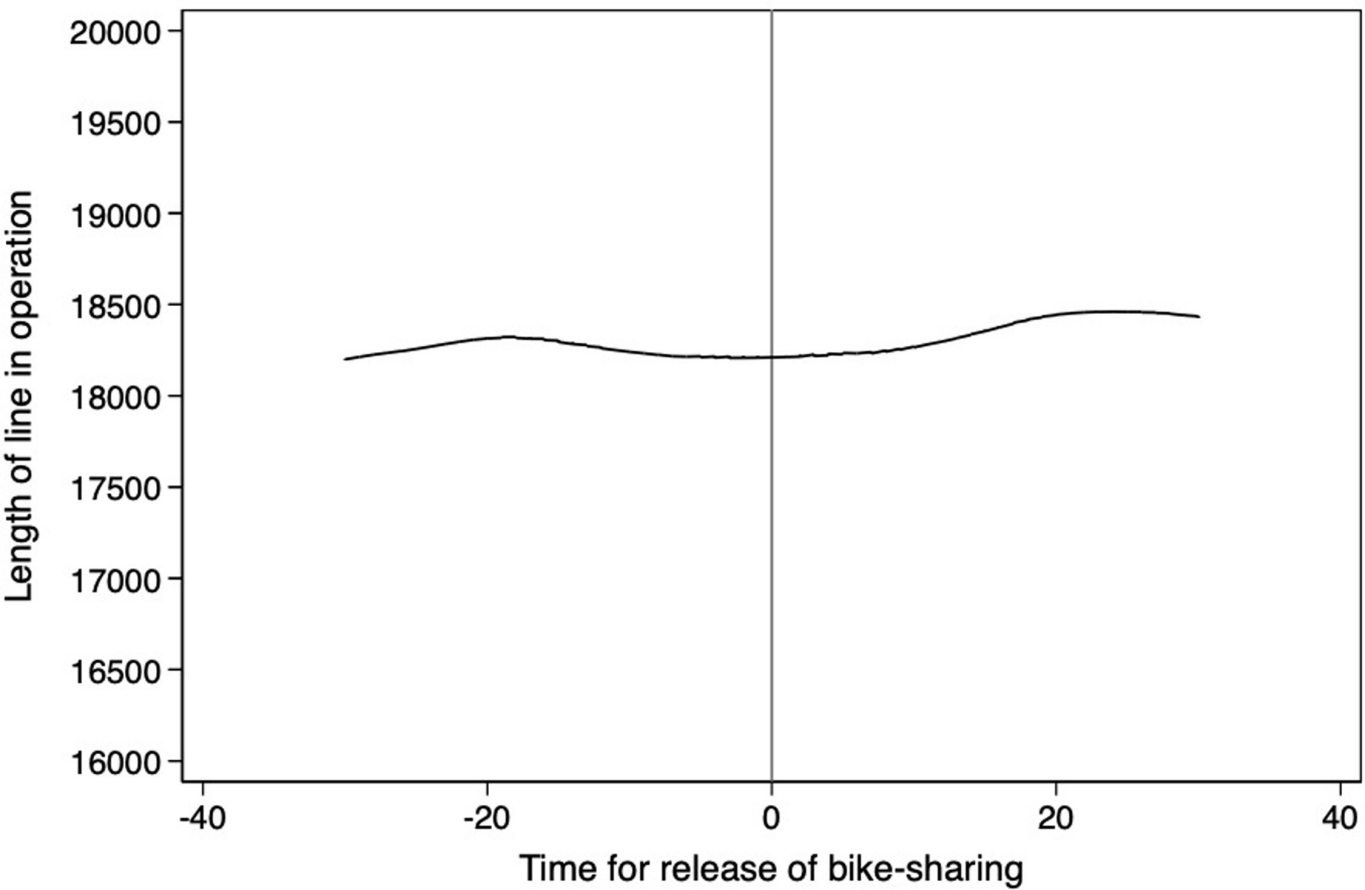
Trends of length of subway and bus lines in operation for 25 days before and after the launch of new bike-sharing services.

**Fig 8:**
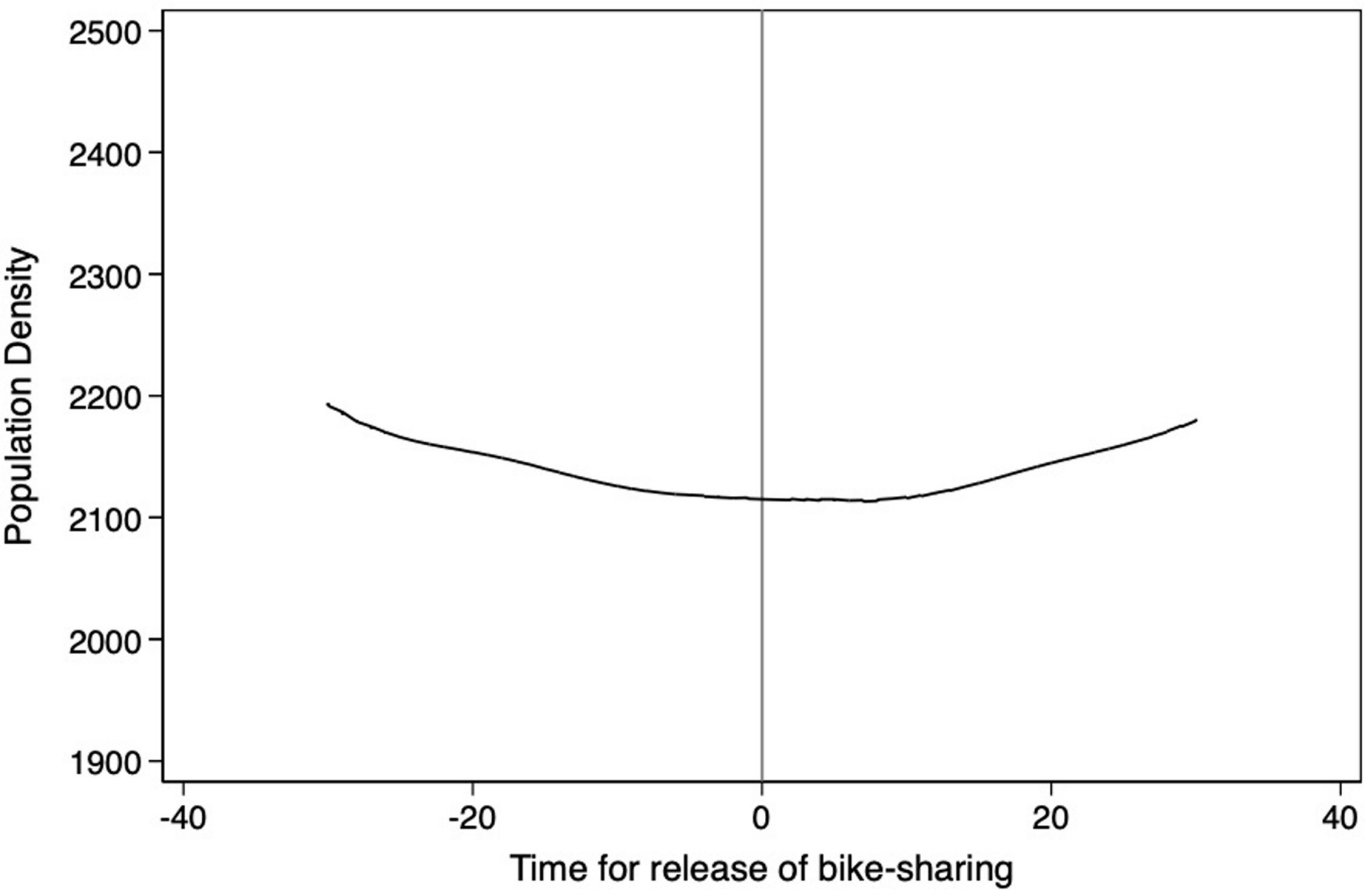
Trends of population density for 25 days before and after the launch of new bike-sharing services.

**Figs 4**–**8** support the above assumption by plotting the trends of traffic restrictions, extreme weather, DNvalue, length of subway and bus lines in operation, and population density for the newly launched bike-sharing services 25 days before and after their opening. We see no discontinuity during the bike-sharing opening dates across these periods. In the **S1 Appendix**, we use traffic restrictions, extreme weather, DNvalue, length of subway and bus lines in operation, and population density as explained variables to test whether they are continuous at the breakpoint. The estimation results for the second- and third-order polynomials in **Table A1 of S1 Appendix** support the validity of our empirical findings.

#### 4.3.6 Extreme value test

To examine the effects of outliers in the CDI distribution, we winsorize the CDI distribution at the 1% and 99% points of the distribution. We report the baseline results in **Table 7**. Our results remain robust.

**Table 7:** Extreme value test.

Dependent variable:	CDI		
	(1)	(2)	(3)
	Linear	Binomial	Trinomial
**Bikesharing**	-0.023	-0.132^**^	-0.226^***^
	(0.038)	(0.056)	(0.068)
Restriction	Y	Y	Y
Extreme Weather	Y	Y	Y
DNvalue	Y	Y	Y
Population Density	Y	Y	Y
Length of line in operation	Y	Y	Y
Observations	463	463	463

Notes: The table covers 25 days before and after the launch of bike-sharing services. We winsorize the CDI distribution at the 1% and 99% points of the distribution. We include week fixed effects in all columns. In columns (1) to (3), we include covariates that may affect travel patterns, namely, the dummies of travel restrictions, extreme weather, weekends, and the control variables of DNvalue, population density, and length of subway and bus lines. Robust standard errors are reported in parentheses; *** p<0.01, ** p<0.05, and * p<0.1.

### 4.4 Heterogeneity analysis

In this section, we examine the relationship between bike-sharing and traffic congestion by grouping bike-sharing services based on their launch date and market share.

#### 4.4.1 Time heterogeneity

Bike-sharing services in China were mainly launched in 2016 and 2017, and only Green Orange Bike was available in 2018. We divide our sample into two groups based on the year. Owing to the limited sample size in 2017, we included the data for 2017 and 2018 in the same group to test the effect of the two groups.

Columns (1) and (2) of **Table 8** report the results for bike-sharing services launched in 2016, whereas columns (3) and (4) report the results for the bike-sharing services introduced from 2017 to 2018. Columns (2) and (4) present the results of interest. Compared with those services launched in 2017 and 2018, the bike-sharing services launched in 2016 have more significant alleviation effects on traffic congestion. A plausible explanation is that the early deployment of these services in 2016 reached the maximum number that the market could support, rendering the subsequent expansions in 2017 and 2018 ineffective.

**Table 8:** Time heterogeneity test.

Dependent variable:	CDI			
	(1)	(2)	(3)	(4)
Bandwidth:	25			
	2016		2017–2018	
	Binomial	Trinomial	Binomial	Trinomial
**Bikesharing**	-0.178**	-0.305***	-0.035	-0.054
	(0.087)	(0.105)	(0.041)	(0.051)
Restriction	Y	Y	Y	Y
Extreme Weather	Y	Y	Y	Y
DNvalue	Y	Y	Y	Y
Population Density	Y	Y	Y	Y
Length of line in operation	Y	Y	Y	Y
Observations	232	232	231	231

Notes: Columns (1) and (2) report the results of the second- and third-order polynomials in 2016, and columns (3) and (4) report the corresponding results in 2016 and 2017. We include week fixed effects in all columns. All columns include extra covariates that may affect travel patterns, including the control variables of DNvalue, population density, and length of subway and bus lines in operation. Robust standard errors are reported in parentheses; *** p<0.01, ** p<0.05, and * p<0.1.

#### 4.4.2 Market shares heterogeneity

We further explore the relationship between bike-sharing and traffic congestion using various subsamples. According to the "*China Bike-Sharing Industry Development Report (2018)*" by *the China Academy of Information and Communications Technology (CAICT)*, based on statistical analysis of indicators such as the number of bikes deployed and app download figures, companies within the industry can be divided into three tiers. The leading bike-sharing businesses include OFO and Mobike, which were followed by Bluegogo, Hellobike, and Youon, with Xiaoming Bike Sharing, U-bicycle, and others trailing behind. Mobike and ofo are undoubtedly leaders in the bike-sharing industry, while third-tier bike-sharing services have fewer users. Therefore, we will only analyze samples from the leading and second-leading brands.

**Table 9** displays the relevant results. The coefficients for the leading bike-sharing services significantly reduced traffic congestion at the 1% significance level. In contrast, the contributions of second-tier bike-sharing firms do not show statistical significance. This evidence indicates that the early deployment of these bike-sharing services had reached the maximum capacity the market could support.

**Table 9:** Heterogeneity test.

Dependent variable:	CDI			
	(1)	(2)	(3)	(4)
Bandwidth:	25			
	the leading ones		the second ones	
	Binomial	Trinomial	Binomial	Trinomial
**Bike sharing**	-0.206**	-0.323***	0.006	-5.4e-05
	(0.083)	(0.1)	(0.045)	(0.056)
Restriction	Y	Y	Y	Y
Extreme Weather	Y	Y	Y	Y
DNvalue	Y	Y	Y	Y
Population Density	Y	Y	Y	Y
Length of line in operation	Y	Y	Y	Y
Observations	263	263	200	200

Notes: Based on their market shares, we divide the bike-sharing services into two groups for analysis. We include week fixed effects in all columns. All columns include extra covariates that may affect travel patterns, including the control variables of DNvalue, population density, and length of subway and bus lines in operation. Robust standard errors are reported in parentheses; *** p<0.01, ** p<0.05, and * p<0.1.

We further test the effects of Mobike and OFO separately (**Table 10**). Columns (2) and (4) present the results of interest. Column (2) shows that the correlation between Mobike and CDI is insignificant, whereas column (4) shows that the introduction of OFO significantly mitigates the average CDI value by 33.602%, which is 2.5 times the baseline regression results. These findings indicate that all bike-sharing services other than OFO have minimal effects on CDI.

**Table 10:** Further heterogeneity tests.

Dependent variable:	CDI			
	(1)	(2)	(3)	(4)
Bandwidth:	25			
	Mobike		OFO	
	Binomial	Trinomial	Binomial	Trinomial
**Bike sharing**	-0.05	-0.044	-0.450***	-0.584***
	(0.051)	(0.054)	(0.139)	(0.174)
Restriction	Y	Y	Y	Y
Extreme Weather	Y	Y	Y	Y
DNvalue	Y	Y	Y	Y
Population Density	Y	Y	Y	Y
Length of line in operation	Y	Y	Y	Y
Observations	132	132	131	131

Notes: Columns (1) and (2) report the results of the second- and third-order polynomials for Mobike, whereas columns (3) and (4) report the corresponding results for OFO. We include week fixed effects in all columns. All columns include extra covariates that may affect travel patterns, including the control variables of DNvalue, population density, and length of subway and bus lines in operation. Robust standard errors are reported in parentheses; *** p<0.01, ** p<0.05, and * p<0.1.

Does this result suggest that the leading companies, especially OFO, have higher operational and management efficiency compared to other companies? In reality, OFO has become a notorious non-performing asset due to poor management among other reasons. This finding does not relate to the brand of bicycles but is more likely a result of an oversaturated bike-sharing market. According to the *2016 China Bike-Sharing Market Research Report*, by the end of that year, OFO had deployed 800,000 bicycles, capturing a market share of 51.2%, while Mobike had released 500,000 bicycles, securing a market share of 40.1%. At this point, the bike-sharing market in China was highly concentrated, with the leading companies holding a combined market share of 91.2%. Tables 8 and 9 show that the first batch of shared bicycles significantly reduced traffic congestion (see Columns 1–2). However, with other bicycles added in 2017, these effects almost disappeared (see Columns 3–4). This indicates that the additional bikes might have caused disorganized parking and worsened congestion, thus offsetting the initial benefits. The reduction in the coefficient with extended bandwidth also supports this conclusion in Table 4.

### 4.5 Mechanism analysis

This section examines the potential mechanisms through which bike-sharing could ameliorate traffic congestion. Bike-sharing serves as a convenient mode of transport that complements public transit, encouraging individuals to opt for public transportation over driving private vehicles by simply stepping out of their homes and using bike-sharing, thereby alleviating road congestion. We manually collected daily ridership data of subway systems for two months before and after the introduction of bike-sharing in three cities, serving as the dependent variable. Dummy variables representing the introduction of bike-sharing in each city were used as explanatory variables. In considering factors that may affect subway ridership, including motor vehicle restrictions, extreme weather conditions, nighttime illumination, population density, track length, holiday dummy variables, and weekly fixed effects, regression was conducted based on the following model:

$$Subway_{it}=\beta_{0}+\beta_{1}\;Bike-sharing_{it}+\lambda X_{it}+\mu_{it}$$ (3)

Formula (3) defines *Bike-sharing* as a binary variable, representing a dummy variable for the initial implementation of bike-sharing programs in three cities. Concurrently, *Subway* refers to the average daily subway ridership in the two months before and after bike-sharing was introduced. Regression results are presented in **Table 11**. According to the results in column (1), the coefficient for *Bike-sharing* implementation over 25 days is 16.458, statistically significant at the 5% level. In column (2), the coefficient for Bike-sharing implementation over 2 months is 15.08, significant at the 1% level. The results indicate that the introduction of bike-sharing in these cities has led to an increase in subway ridership, providing a viable option for integrated multimodal transportation and effectively reducing road congestion.

**Table 11:** Mechanism analysis.

Dependent variable:	subway	
	(1)	(2)
Bandwidth	25	60
**Bike sharing**	16.458**	15.080***
	(7.208)	(4.896)
Restriction	Y	Y
Extreme Weather	Y	Y
DNvalue	Y	Y
Population Density	Y	Y
Length of line in operation	Y	Y
Holiday	Y	Y
Constant	-106.665***	-115.721***
	(16.916)	(10.805)
Observations	456	957
R-squared	0.953	0.952

Notes: Columns (1)-(2) report the changes in subway ridership 25 days and two months after the launch of the bike-sharing program. We include week fixed effects in all columns. All columns include extra covariates that may affect travel patterns, including the control variables of DNvalue, population density, length of subway and bus lines in operation, and Holiday. Robust standard errors are reported in parentheses; *** p<0.01, ** p<0.05, and * p<0.1.

## 5. Conclusions and policy implications

Based on data from Beijing, Shanghai, and Wuhan from 2016 to 2018, this study empirically tests the short-term effects of bike-sharing platforms on urban traffic congestion using RDD, while controlling for weekly fixed effects. The findings suggest that bike-sharing platforms have significantly reduced urban traffic congestion in the short term. The results show a significant reduction of 0.226 in the Congestion Delay Index (CDI), equivalent to 13% of the average CDI value. Heterogeneity analysis indicates that bike-sharing services launched by leading companies in 2016, particularly OFO, significantly alleviated congestion. In contrast, later bike-sharing initiatives had almost no effect on traffic congestion, suggesting that the current mass deployment of bike-sharing has reached the market’s carrying capacity, and newcomers no longer meet the market’s needs. Mechanism tests indicate that bike-sharing can promote a shift from driving to subway use, thereby easing congestion.

Over time, the presence of bike-sharing in cities has sparked widespread discussion among academics and the public about some of the uncivil social phenomena associated with it and its role in alleviating urban traffic congestion. Some studies indicate that the rapid expansion of bike-sharing systems brings new challenges, such as parking congestion [46]. The impact of bike-sharing on overall traffic flow reduction is limited, possibly only shifting rather than eliminating current traffic congestion [44, 45]. Although other literature indicates a positive correlation between the use of bike-sharing and the volume of bus and subway riders [41], it is difficult to demonstrate that bike-sharing plays a synergistic role in improving traffic congestion. The road space freed up by removing cars is quickly replaced by new cars, thus the impact on traffic congestion is not clear [43]. In contrast, by directly using the traffic congestion index as the dependent variable, we find that in the major urban areas of Beijing, Shanghai, and Wuhan, even in the short term after the introduction of dockless bike-sharing into the traffic ecosystem, dockless bike-sharing directly improved traffic congestion and increased subway ridership, complementing the metro system.

The conclusions of this paper provide useful policy insights for the development of the sharing economy. First, our analysis of the causal impact of dockless bike-sharing on traffic congestion provides valuable evidence for policymakers. In the baseline regression, we estimated the congestion effect of the launch of bike-sharing, which can be further combined with congestion cost literature to roughly estimate the social benefits of the dockless bike-sharing program as a reference to further achieve urban emission reduction and increase social welfare. Second, regulate the development of the sharing economy to help achieve emission reduction targets. Our heterogeneity analysis shows that only a few pioneering companies’ deployment of bike-sharing brought significant improvement effects, which may be due to the subsequent excessive deployment of bikes exceeding the market’s carrying capacity under the bike-sharing investment frenzy. As the lack of regulation and credit assurance that causes vicious competition among enterprises. Therefore, government departments should introduce relevant regulatory policies to regulate the development of the sharing economy and guide the providers of bike-sharing to compete healthily. Third, explore the usage scenarios of bike-sharing, and play the synergistic role of "rail transit + bike-sharing" to promote urban low-carbon emission reduction. Our mechanism analysis shows that dockless bike-sharing has the potential to complement the subway significantly. While advocating the use of bike-sharing, we should also actively expand the usage scenarios of bike-sharing, fully play its connecting function, and cooperate with rail transit.

Our research has several limitations. (1) Our study captures only the short-term effects of the bike-sharing system. The RDD estimates we use are localized and may not accurately reflect the treatment effects on units far from the threshold. Expanded bandwidth might disrupt the continuity of control variables and increase the bias in policy estimates. As the bike-sharing craze subsides, whether it’s positive impact on congestion will persist remains to be seen. (2) Focusing only on three major cities in China limits our comprehensive understanding of the country. Chinese cities vary significantly in natural and economic scale, and future research could further expand and compare findings, potentially providing insightful discoveries. (3) We were unable to obtain accurate deployment data for bike-sharing. The operational data of bike-sharing is usually proprietary to these companies. Lacking data makes it difficult to further ascertain causal relationships. (4) We explored this only for bike sharing, and in fact, our analysis can also be applied to other shared economy-derived business models, such as car sharing. Future research could expand to compare the impacts of these sharing methods on urban development.

## Supporting information

S1 Dataset (XLS)

S1 Appendix Placebo tests for continuity of control variables. (DOCX)
