# Supplementary material for: Does bike-sharing reduce traffic congestion? Evidence from three mega-cities in China
Source: PLoS One. 2024 Aug 20;19(8):e0306317. doi: 10.1371/journal.pone.0306317 (PMC11335155; doi:10.1371/journal.pone.0306317)
Supplement: S1 Appendix — (DOCX) [file pone.0306317.s002.docx]

Appendix. Placebo tests for Continuity of control variables

In **Table A1**, we use traffic restrictions, extreme weather, DNvalue, the length of subway and bus lines in operation, population density, and a unicentric city as the explained variables to test whether they are continuous at the breakpoint. We list the estimation for the second- and third-order polynomials in Appendix **panels A** and **B**.

**Table A1. Continuity of control variables**

| Bandwidth: | 25 | | | | | |
| --- | --- | --- | --- | --- | --- | --- |
| Panel A |  |  |  |  |  |  |
|  | (1) | (2) | (3) | (4) | (5) | (6) |
| Dependent variable: | **Restriction** | | **Extreme Weather** | | **DNvalue** | |
|  | Binomial | Trinomial | Binomial | Trinomial | Binomial | Trinomial |
| **Bike sharing** | 0.300* | 0.228 | 0.016 | -0.21 | -0.017 | 0.188 |
|  | (0.141) | (0.196) | (0.138) | (0.187) | (1.09) | (1.469) |
| Observations | 463 | 463 | 463 | 463 | 463 | 463 |
| Panel B |  |  |  |  |  |  |
|  | (7) | (8) | (9) | (10) |  |  |
| Dependent variable: | **Length of line in operation** | | **Population Density** | | |  |
|  | Binomial | Trinomial | Binomial | Trinomial |  |  |
| **Bike sharing** | -8.88 | 417.504 | -2.58 | 52.853 |  |  |
|  | (1937.515) | (2616.191) | (360.445) | (486.838) |  |  |
| Observations | 463 | 463 | 463 | 463 |  |  |

Notes: Robust standard errors are reported in parentheses; *** p<0.01, ** p< 0.05, and * p <0.1.
